# Collagen Kinase Receptors as Potential Therapeutic Targets in Metastatic Colon Cancer

**DOI:** 10.3389/fonc.2020.00125

**Published:** 2020-02-12

**Authors:** Marie Lafitte, Audrey Sirvent, Serge Roche

**Affiliations:** CRBM, CNRS, Univ. Montpellier, Montpellier, France

**Keywords:** collagen, extracellular matrix, tumor microenvironment, receptor, tyrosine kinase, colorectal cancer, metastasis, targeted therapy

## Abstract

Colorectal cancer (CRC) is one of the leading causes of tumor-related death worldwide. While surgery can cure patients with early stage CRC, the 5-year survival rate is only 10% for patients with metastatic disease. Therefore, new anti-metastatic therapies are needed for this cancer. Metastatic spread defines the dissemination of cancer cells with tumor-initiating capacities from the primary tumor and their colonization of distinct organs, mainly the liver, for secondary tumor formation. Although the underlying mechanisms are not fully understood, components of the tumor microenvironment have gained strong interest. Among the known metastatic-promoting factors, collagens are extracellular matrix components that are deposited within the tumor, the tumor microenvironment, and at metastatic site(s), and are recognized to play essential roles during metastasis development. Here, we review recent findings on the metastatic role of the collagen receptors Discoidin Domain Receptors 1 and 2 (DDR1 and DDR2) in CRC and discuss the therapeutic value of targeting these receptor tyrosine kinases in this cancer.

## Introduction

Colorectal cancer (CRC) remains one of the leading causes of malignancy-related death worldwide. While early-stage tumors have good prognosis, the 5-year survival rate is lower than 10% for patients with metastatic CRC (1). CRCs are heterogeneous in nature and their development is influenced by specific genetic, epigenetic, and environmental factors (1). The molecular characterization of CRC for therapeutic decision-making has identified four consensus molecular subtypes (CMS 1-4) (2). CMS1 represents hyper-mutated, microsatellite instable (MSI+) tumors with strong immune activation; CMS2 are WNT/MYC-dependent proliferative tumors; CMS3 include KRAS-mutated tumors and tumors with dysregulated metabolism; and CMS4 tumors are characterized by strong stromal infiltration. Targeted therapies have been developed for metastatic CRC (mCRC), but they display moderate clinical effects. For instance, anti-EGFR or -VEGFR agents prolong patient survival by only few months. Moreover, anti-EGFR therapies cannot be used for KRAS-mutated CRC because of systematic innate resistance (3, 4). Similarly, the results obtained with immune checkpoint inhibitors, such as anti-Programmed cell Death 1 (PD1) antibodies, are variable due to poor immune infiltration, except in the CMS1 subtype (5, 6). Currently, effective therapies for mCRC remain a challenge.

## Collagens in CRC Metastases

CRC metastatic spread is characterized by dissemination of specific tumor cell clones with tumor-initiating properties primarily to the liver due to venous drainage (7). The underlying molecular causes are not well-known, but they might not involve additional genetic alterations (7). Indeed, CRC dissemination seems to be an early event (i.e., metastatic clones have disseminated before the tumor clinical detection) (8, 9). Metastasis development may be mainly influenced by aberrant tumor cell communication with specific components of the tumor microenvironment, the immune system, the blood circulation, or the metastatic niche, in line with the seed and soil theory originally formulated by Paget (7, 10). Among the metastatic factors involved in this process, extracellular matrix (ECM) components have gained strong interest. Specifically, collagens, which are the most abundant ECM components, have been involved in tumor progression (7, 10). Aberrant collagen I, IV, and XVII protein levels in CRC samples have been associated with worse prognosis and metastasis development (11, 12). Collagens are produced by cancer-associated fibroblasts (CAF), tumor-associated macrophages (TAM) and tumor cells, and are deposited within or around the tumor or at the metastatic niche, mostly via cancer exosomes, and TAMs (13, 14). Collagen deposition induces tumor stiffness, resulting in enhanced tumor growth, reduced immune infiltration, and metastatic colonization (12, 15). Besides their type, the level of collagen architecture (i.e., polymerization, fiber alignment, and distribution) also might influence metastatic progression. Mounting evidences indicate that dense and aligned collagen fibers favor cancer cell invasion (16, 17). Enzymatic remodeling of collagen polymers also is involved in this malignant process. Specifically, well-known collagen modifiers expressed by tumor or stromal cells, such as metalloproteases, collagenases and lysine oxidases, influence collagen architecture by promoting cross-linkage and stabilization of insoluble collagen deposited in tumor tissues, thus enabling CRC progression (11, 12, 18). Mechanistically, accumulation of collagen fibers induces an integrin-dependent mechanotransduction pathway that involves actin cytoskeleton contraction (19, 20). Other post-translational modifications of the collagen matrix might contribute to their metastasis-promoting effect, as recently evidenced for Peptidyl Arginine Deaminase 4 (PAD4) (21). Specifically, PAD packed in tumor-derived exosomes increases the stiffness of collagen fibers deposited in the liver pre-metastatic niche, through conversion of arginine residues into citrullin residues. Stiffened collagen matrix increases the adhesion of CRC cells at the metastatic site, promoting mesenchymal to epithelial transition, and enabling liver metastasis growth.

## The Collagen Receptors DDR1 and DDR2

The many different collagen entities detected in the tumor microenvironment suggest the existence of complex, not-yet fully characterized mechanisms that influence tumor progression. For instance, it was suggested that integrins mediate tumor signaling induced by highly cross-linked collagen fibers (22), while the tumor-promoting effects of soluble fibrillar collagens are independent from integrin engagement (23). This tumor-promoting activity might be mediated by a poorly characterized class of collagen receptors called Discoidin Domain Receptors (DDR) (24, 25). DDRs include DDR1 and DDR2 and belong to the receptor tyrosine kinase family (RTK) (24, 25). They are evolutionarily conserved, but they are distinct from the other RTKs due to their capacity to bind to ECM components (26, 27). DDR1 and DDR2 share highly conserved sequences and a similar modular structure (i.e., extracellular domain with binding affinities to collagens, short transmembrane domain, and large cytoplasmic tail containing a kinase domain), but they differ in collagen binding, tissue expression, and signaling. Indeed, DDR1 is activated by most collagen types, including I and IV, which is abundant in the basement membrane. Conversely, DDR2 is only activated by fibrillary collagens, specifically collagen I, III, and X (24, 25). DDR1 is preferentially expressed in epithelial tissues, whereas DDR2 is expressed in mesenchymal tissues (24, 25). Unlike other RTKs, DDR activation kinetic is slow (detected after 1 h of collagen stimulation), but sustained over time (more than 1 day). Although the underlying mechanism is not fully understood, it has been proposed that collagen induces the lateral association of DDR1 dimers (i.e., receptor clustering) and phosphorylation between dimers (28–30). Whether DDR2 is activated through a similar mechanism remains unclear (30). Indeed, it was reported that DDR2 activation can be mediated by Src-induced phosphorylation of its activation loop (31, 32). DDRs act as a cellular sensor of the ECM microenvironment and can cross-talk with several transmembrane receptors, such as Notch, TGF-β and adhesive receptors, and influence their signaling activity upon collagen deposition (23, 33). In physiological conditions, DDRs regulate cell polarity, adhesion, migration, and proliferation. Knock-out mice showed that DDR1 has a role in mouse mammary gland development, specifically in stromal-epithelial interaction during ductal morphogenesis (34), and that DDR2 acts as an ECM sensor to modulate cell proliferation, required for bone formation (35). However, it is not known whether DDRs have a role in intestinal epithelium development and homeostasis.

## DDR1 in CRC Metastases

**Figure 1:**
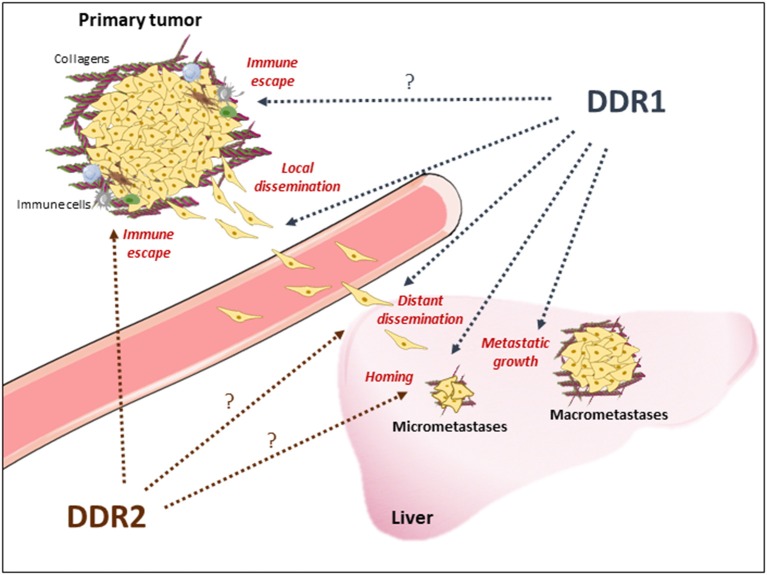
Proposed DDR1 and DDR2 functions during metastasis development of CRC. DDR1 and DDR2 activation upon collagen deposition may promote local CRC cell invasion from the primary tumor, through invadosomes formation and epithelial cell migration, and immune evasion enabling cell dissemination; CRC cells lending at the metastatic site for CRC cells survival; metastatic reactivation (micrometastases) and development (macrometastases). Note that DDRs functions reported in other tumor-types and to be confirmed in CRC are indicated with a question-mark. Immune cells and collagens deposition around the tumor or at the metastatic niche are indicated.

DDR1 oncogenic role in human cancers was first highlighted by global phospho-tyrosine profiling in lung cancer (36). Since then, many evidences of an important DDR1 tumor-promoting role in metastasis development have been reported, although this activity may depend on the tumor type and the collagen microenvironment nature. For instance, DDR1 has been involved in the collective migration of squamous cell carcinoma (37) and breast tumor cells (38), metastatic reactivation in breast cancer (23), homing and colonization of lung and bones (23, 39), and peritoneal metastases from gastric carcinoma (40). Moreover, in lung cancer, KRAS mutations induce DDR1 expression to sustain tumorigenesis (40). We and others (41, 42) recently showed that DDR1 promotes CRC cell invasion and metastatic behavior in nude mice, and that its overexpression potentiates these properties. DDR1 also regulates invasiveness of patient-derived cell lines from mCRC and circulating CRC cells, which are at the origin of metastasis development (42). These studies also suggest that DDR1 acts at different steps of CRC liver metastasis formation (Figure 1). First, *in vitro* evidence support DDR1 role in local invasion by primary tumor cells and in the invasive properties of disseminated CRC cells, which is essential for metastasis formation. DDR1 activity may then promote CRC cell homing in the liver upon collagen deposition (Figure 1). Finally, DDR1 inhibition displays anti-tumor activity in mice that have already developed DDR1-dependent metastatic nodules, revealing an additional important DDR1 role in metastatic growth (42). Consistently, DDR1 expression level is associated with shorter overall survival in patients with mCRC, and DDR1 phosphorylation is strongly increased in the corresponding metastatic lesions (42, 43). Interestingly, DDR1 upregulation is an independent marker of poor prognosis in patients with stage IV CRC, and is not correlated with any CMS subtype (42). How DDR1 oncogenic activity is induced in human cancer is not clear, because DDR1 is not frequently mutated. DDR1 upregulation has been linked to oncogenic activation, such as KRAS mutations (44), a collagen-dependent amplification loop mechanism, and epigenetic mechanisms. Although all these mechanisms may contribute to DDR1 aberrant expression in CRC, a miRNA-dependent epigenetic mechanism was recently documented in this cancer (41, 45).

Several kinase-dependent and kinase-independent mechanisms by which DDR1 promotes metastatic progression have been reported, depending on the tumor type and/or the stage of metastasis development. For instance, DDR1 activates, via a kinase-independent mechanism, Tuba and CDC42 to induce early proteolysis-based invasion of breast tumor cells (38). By interacting with the tetraspanin TM4SF1, DDR1 recruits PKC alpha to activate JAK2, leading to STAT3 activation for metastatic reactivation (23). Conversely, bladder tumor cells colonize airway smooth muscle cells, a rich source of collagen III in lung, via a DDR1 kinase-dependent mechanism, leading to STAT3 transcriptional activation (46). Similarly, DDR1 kinase activity is required for K-RAS-driven lung cancer and Notch tumor signaling (44). In CRC, we established the central role of DDR1 kinase activity in metastatic progression, as indicated by the loss of such function upon introduction of a kinase-inactive mutation or pharmacological inhibition (42). By phospho-proteomic analysis of tyrosine phosphorylation, we then revealed that DDR1 acts through a Wnt/β-catenin-dependent and RAS-independent mechanism. Specifically, we identified two unsuspected DDR1 substrates involved in this oncogenic process: the signaling protein Breakpoint Cluster Region (BCR) and the pseudo-kinase PEAK1 of the Pragmin family (42, 47). Mechanistically, DDR1 phosphorylation of BCR on tyrosine 177 alleviates a negative regulatory loop on β-catenin signaling to sustain its oncogenic activity, resulting in the induction of genes that are important for tumor cell dissemination and metastasis development, such as *MYC, CYCD1*, and *LGR5* (42, 48). Although not investigated in this study, DDR1 may also induce PEAK1 invasive activity (49, 50), possibly via a YAP1-dependent mechanism, as recently suggested (51). As nuclear YAP1 can form a β-catenin transcription complex that is essential for the transformation and survival of β-catenin-driven cancer (52), we propose that DDR1 supports metastatic development in a collagen-rich environment via a BCR- and PEAK1-dependent mechanism.

## DDR2 in CRC Metastases

The first evidence of DDR2 oncogenic role in human cancer came from its alteration in squamous lung cancer (53). Afterwards, DDR2 was found to be upregulated in many epithelial malignancies, including breast (54) and ovarian tumors (55), and plays a major role in epithelial to mesenchyme transition (EMT) and metastasis development (54, 55). Mechanistically, DDR2 activity stabilizes the transcription factor and EMT inducer SNA1 (54). DDR2 upregulation in the stroma also may participate in this malignant process by promoting tumor stiffness through integrin-mediated mechanotransduction in CAFs and by promoting stromal-breast cancer cell interaction for metastatic colonization (56–58). Interestingly, these DDR2 oncogenic activities require a Src-dependent kinase activation mechanism (54). In CRC, evidence for similar DDR2 tumor-promoting functions is lacking. Nevertheless, a recent report suggested that epithelial DDR2 could participate in metastatic progression (Figure 1). Specifically, in a small cohort of patients with CRC, DDR2 level in tumors was associated with high frequency of peritoneal dissemination and poor prognosis (59). It is unclear whether stromal DDR2 has a similar metastatic role in CRC as in breast tumors. A mouse study suggested that stromal DDR2 deficiency predisposes the hepatic tissue to CRC metastases (60) by fostering trans-differentiation of hepatic stellate cells into myo-fibroblasts for metastatic niche development (60). Whether a similar mechanism operates in human CRC is unknown. Finally, an *in vivo* functional genomic study using isogenic mouse cancer models to identify genes the inhibition of which potentiates the response to anti-PD1 immunotherapy showed that tumor DDR2 is an essential regulator of MSI+ CRC cell immune evasion (Figure 1) (61). Whether DDR2 has a similar role in microsatellite-stable CRC cells remains to be tested. Similarly, it was suggested that DDR1 promotes breast tumor growth by suppressing the anti-tumor immunity (62). How exactly and in which circumstances DDR1 and DDR2 may regulate human tumor evasion, particularly in CRC, deserve further investigation.

## Targeting DDR Tumor Activity in Metastatic CRC

All these results suggest that DDR1 and possibly DDR2 are attractive therapeutic targets in mCRC. DDR inhibition could reduce metastasis dissemination or reactivation, and prevent disease relapse (Figure 1). This therapeutic strategy may be particularly relevant for tumors that disseminate at an early stage, as recently suggested for CRC. Moreover, DDR inhibition could reduce metastatic growth, thus facilitating metastatic nodule resection, and also sensitize “cold” tumors to immune checkpoint-based therapies. The fact that DDR1 expression level is not restricted to any specific CMS subclass and that its tumor-promoting function is KRAS mutation-independent suggests that DDR1 inhibitors could be active in all CRC subtypes, including CMS3 tumors for which the therapeutic options are limited. As DDR1 tumor-promoting function in CRC requires its kinase activity, small DDR1 kinase inhibitors might be of therapeutic value. Interestingly, chemical proteomic profiling of several clinical TK inhibitors, including those targeting oncogenic Src or ABL activities, identified DDRs as additional major targets. For instance, DDR1 and DDR2 are inhibited by the anti-leukemic agents nilotinib, bosutinib, and dasatinib (IC_50_ in the nM range) (Table 1) (67, 74, 75). This important observation suggests that DDR inhibition may contribute to the clinical effects of these compounds, and that these inhibitors could be used to target DDR-dependent tumors, including mCRC. We validated this second hypothesis in a preclinical model by showing a strong anti-metastatic activity of nilotinib in DDR1-dependent mCRC cells (42). The major DDR1 role in this response was demonstrated by the lack of nilotinib activity in CRC cells that express a kinase-dead DDR1 mutant. Similarly, targeting DDR2 activity with dasatinib enhanced the tumor response to anti-PD1 immunotherapy in a CRC mouse model (Table 1) (61). Overall, these results predict that these anti-leukemic agents have also an anti-CRC effect. They could be combined with immune checkpoint inhibitors, particularly in tumors with high DDR level/activity. More recently, several ATP-site inhibitors have been developed to specifically inhibit DDR1 and/or DDR2 activity, and they display significant anti-tumor activities in several cancer models, including CRC cells (Table 1) (69, 71, 76). As these receptors can also signal through kinase-independent mechanisms, non-kinase inhibitors have been developed to target these tumor-promoting activities. For instance, anti-DDR1 antibodies can interfere with DDR1 binding to collagens, by sterically blocking the extracellular association of DDR1 subunits (Table 1) (73). Similarly, a neutralizing antibody against DDR1 inhibits breast tumor growth in a mouse model by suppressing the anti-tumor immunity (62). Due to DDR1 aberrant expression in CRC, an anti-DDR1 antibody-drug conjugate was recently developed for CRC treatment. This agent displayed significant anti-tumor activity in a preclinical model of CRC, without overt toxicity in control animals (Table 1) (43). Finally, small-molecule allosteric inhibitors of DDR2 extracellular domain inhibit the tumor–microenvironment interaction and breast tumor invasion (70). Whether such inhibitor displays similar anti-invasive effect in CRC was not reported.

**Table 1:** Anti-tumor activity of DDRs inhibitors/antibodies in CRC.

	**molecule**	**IC_**50**_ DDR1 (nM)**	**IC_**50**_ DDR2 (nM)**	**Biological effects in CRC**	**References**
Multi-kinase inhibitor	Dasatinib (BMS-354825)	0.5	1.4	Enhances the anti-tumor response of anti-PD1 in a CRC mouse model	(61, 63)
	Imatinib (STI571)	337	675	Inhibits CRC cell growth and stromal-induced growth stimulation	(63, 64)
	Nilotinib (AMN107)	43	55	Inhibits CRC cells invasion and metastatic development in nude mice	(42, 63)
	Ponatinib (AP24534)	9	9	Inhibits CRC cell migration Inhibits CRC tumor growth in nude mice	(65, 66)
	Bafetinib (INNO-406)	n/a	220	n/a	(67)
	Sitravatinib (MGCD516)	29	0,5	n/a	(68)
DDRs kinase inhibitor	Compound 1	10	234	n/a	(69)
	Compound 2	21	76	n/a	(69)
	Compound 4	279	162	n/a	(69)
	WRG-28	–	230	n/a	(70)
	DDR1-IN-1	105	413	Inhibits CRC cells growth	(71)
	DDR1-IN-2	47	143	Inhibits CRC cells growth	(71)
	7rh	6,8	101,4	n/a	(72)
	7rj	7	93,6	n/a	(72)
DDR1 antibody	T4H11-DM4 antibody	n/a	–	Inhibits CRC tumor growth in nude mice	(43)
	mAb 3E3	n/a	–	n/a	(73)
	Neutralizing DDR1 antibody	n/a	–	n/a	(62)

## Conclusion and Future Directions

Since their discovery more than 20 years ago, the DDR1 and DDR2 collagen receptors are considered critical regulators of cancer invasion. Specifically, they may promote important cancer functions in collagen-rich microenvironments (i.e., cell survival, invasion, cancer stem cell traits, and immune evasion) that are required for mCRC development. As a result, these receptors are becoming attractive therapeutic targets in CRC (77). However, many important questions remain to be addressed to better understand their roles in CRC and to successfully develop anti-metastatic therapies targeting DDR signaling. First, it will be important to clarify DDR1 and DDR2 respective roles in CRC, specifically in the stromal and tumor compartments. Moreover, as development pathways are often reactivated in cancer, it would be important to address their physiological roles in intestinal homeostasis and regeneration. Due to the complexity of DDR signaling, any kinase-independent function in CRC should be explored because it could have important therapeutic consequence. Similarly, much research is needed to describe the largely unknown DDR1 and DDR2 kinase regulation, and its deregulation in CRC. Although DDR1 upregulation and aberrant tumor collagen deposition are obvious mechanisms, additional mechanisms may be expected. How DDRs induce cancer signaling is another critical question, although we established an important connection between DDR1 signaling and the β-catenin pathway (42). Last, but not least, recent reports uncovered unsuspected DDR roles in CRC immune evasion (61, 62). How these receptors contribute to this cancer hallmark is a basic and clinical question because DDR signaling inhibition could define a therapeutic strategy to reduce metastatic development and sensitize CRC to immune checkpoint inhibitors.

## Author Contributions

SR drafted the first version of the manuscript. ML and AS contributed towards the figure and the table. All authors have critically reviewed and approved the manuscript.

### Conflict of Interest

The authors declare that the research was conducted in the absence of any commercial or financial relationships that could be construed as a potential conflict of interest.
